# Active tuberculosis screening among the displaced population fleeing Ukraine, France, February to October 2022

**DOI:** 10.2807/1560-7917.ES.2023.28.12.2300155

**Published:** 2023-03-23

**Authors:** Jean-Paul Guthmann, Philippe Fraisse, Isabelle Bonnet, Jérôme Robert

**Affiliations:** 1 Santé publique France, Saint Maurice, France; 2 Réseau National des Centres de lutte antituberculeuse (CLAT), Strasbourg, and Groupe pour la recherche et l’enseignement en pneumo-infectiologie de la Société de pneumologie de langue française, Paris, France; 3 Centre National de Référence des Mycobactéries, Laboratoire de Bactériologie-Hygiène, Site Pitié-Salpêtrière, Groupe hospitalo-universitaire APHP, Sorbonne Université, and CIMI-Paris, Inserm U1135, Sorbonne Université, Paris, France

**Keywords:** tuberculosis, active screening, displaced, France

## Abstract

Persons fleeing Ukraine since February 2022 have potentially higher risk of tuberculosis (TB) vs all European Union countries. Interest of active TB screening among this population is debated and not widely adopted. In this screening intervention by a network of TB centres in France, the number needed to screen (NNS) was 862 to find one case. This experience shows that this strategy may be relevant for TB control in situations of massive displacement, similar to that following the Russian invasion.

On 20 March 2022, the tuberculosis (TB) control network in France (Centres de lutte antituberculeuse (CLAT)) issued a warning regarding health issues, including TB, of persons arriving from Ukraine following the Russian invasion in February 2022. We sought to evaluate the results of the French active screening strategy based on an existing network of TB centres to detect TB in people coming from Ukraine to France.

## Tuberculosis screening of migrants in France

Following the Russian invasion of Ukraine on 24 February 2022, the Council of the European Union (EU) adopted a decision on the temporary protection of persons fleeing Ukraine. This emergency mechanism, which aimed to provide an immediate and large protection to displaced persons [1], was implemented in France on 10 March 2022 [2]. Because Ukraine reported the second highest number of TB cases in the World Health Organization (WHO) European Region (incidence rate: 65/100,000 population) in 2019 and 27% of multidrug-resistant (MDR) TB among new cases [3], a large population movement from this country may thus challenge and put national TB programmes under high pressure [4]. Hence, the district authorities and practitioners were reminded of the French guidelines regarding the screening and management of migrants arriving from a high TB incidence country, on 23 March 2022 [5]. These include the possibility of a systematic full health consultation within 4 months after arrival [6] and a chest X-ray (CXR) for TB screening [7]. 

In metropolitan France, active TB screening is usually performed by the 105 CLATs located in each of the 96 districts, and less frequently by outreach screening campaigns in schools, camps or shelters for migrant workers [8,9]. The staff of the CLAT provided active recruitment for TB screening among the displaced population either from where they lived or stayed temporarily, or in persons that on some occasions were referred for screening by the administrative authority. Active screening for TB was considered when a person had been actively referred to the TB centre and had performed a CXR.

## CLAT screening activities February to October 2022

A questionnaire was administered to the CLAT network by the network coordinator to assess the number of TB cases recorded among people fleeing from Ukraine and the number of persons screened during the first 8-month period (February–October 2022) after the Russian invasion.

Data from this survey (number of persons screened, characteristics and the context of diagnosis of each TB case recorded, e.g. through active screening or other) were collected by mail or by phone call to the CLAT staff. All 105 TB centres responded to the survey. A total of 17 TB cases had been recorded in displaced persons from Ukraine during the survey period, including three persons (one adult aged ca 60 years, one 8-month-old child and a young adult from a country in central Africa residing in Ukraine) who had TB treatment interrupted after fleeing the country. The 17 cases had a median age of 42 years (range: 8 months–65 years) and the male/female sex ratio was 1.12. Six cases were confirmed as MDR by the National reference laboratory (NRL) for Mycobacteria (none were extensively drug-resistant (XDR)) (Table 1). Among the 17 TB cases, 10 were identified through active screening among a total population of 8,621 screened persons (Table 2), resulting in a TB prevalence of 116 cases per 100,000 population (i.e. 10/8,621). Consequently, the number needed to screen (NNS), i.e. the inverse of the prevalence of TB in the screened population [10], was 862 persons screened to find one TB case. Among the 10 cases identified through active screening, there were nine Ukrainians and one person from a high MDR TB burden country in the WHO European Region, seven were non-symptomatic and four were MDR (Table 1).

**Table 1:** Characteristics of tuberculosis cases in persons fleeing from Ukraine reported by CLAT network, France, February–October 2022^a^ (n = 17)

Screening vs other	Case	Context of diagnosis	Symptoms	Month of screening/visit	Country or region of birth	Clinical Site	Microscopy (AFB)	Culture	MDR
Screening	1	Active screening	Yes	Mar	Ukraine	Pulmonary	Negative	Positive	Yes
	2	Active screening	Yes	May	Ukraine	Pulmonary	Positive	Positive	No
	3	Active screening	No	May	Ukraine	Pulmonary	Negative	Positive	No
	4	Active screening	No	Jun	Ukraine	Pulmonary	Positive	Positive	No
	5	Active screening	No	July	Ukraine	Pulmonary	Positive	Positive	No
	6	Active screening	No	Aug	Ukraine	Pulmonary	Negative	Positive	Yes
	7	Active screening	Yes	Sep	Eastern Europe	Pulmonary	Negative	Positive	Yes
	8	Active screening	No	Aug	Ukraine	Pulmonary	Negative	Positive	Yes
	9	Active screening	No	Oct	Ukraine	Pulmonary	Negative	Positive	No
	10	Active screening	No	Oct	Ukraine	Pulmonary	Negative	Negative	No
Other	11	Presented to CLAT for continuation of treatment	No	Mar	Ukraine	Pulmonary	Positive	Negative	No
	12	Presented to CLAT for continuation of treatment	No	Mar	Ukraine	Pulmonary	Positive	Negative	No
	13	Presented to CLAT because of symptoms	Yes	Mar	West Africa	Pulmonary/bone	Positive	Positive	No
	14	Presented to CLAT because of symptoms	Yes	Jul	Ukraine	Pulmonary	Positive	Positive	Yes
	15	Presented to CLAT because of symptoms; TB treatment discontinued in October 2021	Yes	Jun	Central Africa	Pulmonary	Negative	Positive	No
	16	Referred to CLAT by a general practitioner	Yes	Jul	Ukraine	Pulmonary	Negative	Positive	Yes
	17	Contact investigation, family member of Case 12	No	May	Ukraine	Pulmonary	Negative	Positive	No

AFB: acid-fast bacillus; CLAT: tuberculosis centre; MDR: multidrug-resistant; TB: tuberculosis.

^a^ All cases arrived to France in 2022.

**Table 2:** Number of tuberculosis cases in persons fleeing from Ukraine reported by the CLAT network by mainland regions and diagnosis context, France, February–October 2022 (n = 17)

Region	Cases detected through active screening by the CLAT		Cases recorded by the CLAT in a context other than active screening				Total cases
	Screened	Cases	Presented to CLAT for continuation of treatment	Presented to CLAT because of symptoms	Referred to CLAT by GP	Contact investigation	
Auvergne-Rhône-Alpes	1,467	3	0	0	0	0	3
Bourgogne-Franche-Comté	461	1	0	0	0	0	1
Bretagne	169	0	1	2	1	1	5
Centre-Val de Loire	1,117	1	0	0	0	0	1
Corse	97	0	0	0	0	0	0
Grand-Est	1,473	0	0	0	0	0	0
Hauts-de-France	50	0	0	0	0	0	0
Ile-de-France	160	0	0	0	0	0	0
Normandie	771	0	0	0	0	0	0
Nouvelle-Aquitaine	926	2	1	0	0	0	3
Occitanie	1,059	3	0	0	0	0	3
Pays de la Loire	490	0	0	0	0	0	0
Provence-Alpes-Côte d’Azur	381	0	0	1	0	0	1
TOTAL	8,621	10	2	3	1	1	17

CLAT: tuberculosis centre; GP: general practitioner.

Of interest, the TB centre of the city of Strasbourg, close to the German border (one of the three main reception centres in France), provided additional data on persons from Ukraine. The main characteristics of the 874 persons who were contacted and offered screening from March to July 2022 were: median delay since arrival: 2.5 months; 67% were female (males, n = 286; females, n = 588); mean age: 29.5 (males) and 33.0 (females) years, p = 0.02; overall range: 8 months–88 years). Out of these, 432 (49%) accepted to be screened by CXR and no TB case was diagnosed. The complete results of this survey will be published at a later occasion.

## Analysis of data provided by the tuberculosis mandatory notification system

To complement the results of the active screening performed by the CLAT network, we analysed data regarding TB among Ukrainians as recorded in the TB mandatory notification system. In this national surveillance, information on TB cases diagnosed in France is entered by physicians in an electronic database on a continuous basis and analysed yearly by Santé publique France [11]. In 2022, 33 new TB cases born in Ukraine were notified through this surveillance system, including all nine cases born in Ukraine who were identified through active screening by the CLAT network (Table 1). Nine of these 33 cases were confirmed by the NRL as MDR-TB (Table 3). The remaining 24 TB cases (including 15 who arrived in France in 2022 after the Russian invasion) were identified through different means, mainly by standard consultation, and not by active screening. Overall, the 33 cases represented a notable increase in the number of cases reported compared with the previous years. Between 2008 and 2019, 55 cases were recorded in persons born in Ukraine (mean: 4.6 cases/year), the more recent figures being eight, four, and four cases for the years 2019, 2020, and 2021, respectively. Considering that the number of persons originating from Ukraine in 2022 in France included 19,100 permanent residents (source: Insee 2019 [12]) and 118,994 who had arrived in France because of the war [13], the incidence of TB in this population could be estimated to 24 cases per 100,000 population.

**Table 3:** Characteristics of tuberculosis cases reported through mandatory notification in persons born in Ukraine, France, 2022 (n = 33)

Characteristics	Cases n = 33	%
Age		
Median in years (SD)	38 (13.5)	
Sex		
Male	19	57
Female	14	43
Context of diagnosis		
Spontaneous presentation to health structure	18	55
Contact investigation	4	12
Active screening	9	27
Other	2	6
Year of arrival		
Before 2022	5	15
2022	23	70
Unknown	5	15
Previous history of tuberculosis		
Yes	4	12
No	23	70
Unknown	6	18
Clinical site		
Pulmonary only	29	88
Extra-pulmonary	2	6
Pulmonary and other	2	6
Microscopy		
Positive	19	57
Negative	12	36
Unknown	2	7
Culture		
Positive	28	85
Negative	5	15
Multidrug-resistant tuberculosis		
Yes	9	27
No	24	73

## Discussion

Our data suggest that active CXR screening was an effective intervention in the French context to detect TB cases among the population displaced from Ukraine. Indeed, 10 cases were detected by the CLAT through active CXR screening, representing more than half of the cases reported by the CLAT during the same period. These 10 cases had all recently left Ukraine as consequence of the war and arrived in France in 2022. Among these 10 cases, seven were asymptomatic cases who would possibly have remained undiagnosed should the screening not have been performed. The relevance of active screening is further reinforced by two factors: (i) three patients were smear positive and hence potentially highly contagious and (ii) four carried an MDR strain, suggesting they represented a particular risk for people living close to them, and early identification allowed a quick implementation of appropriate treatment. We therefore think that active screening was useful regarding TB control, and especially to prevent the occurrence of new and potentially severe cases, pending the effective treatment of detected cases. Our findings are in line with what was found in asylum seekers entering Germany in 2015 where TB cases identified through active screening represented 21% of all cases notified that year [14].

The prevalence of 116 cases per 100,000 population reported by the CLAT network through active screening of the displaced population fleeing Ukraine is higher than the mean prevalence reported in Ukraine [3]. It is also much higher than the figure estimated through the mandatory notification system in France, although this figure may be underestimated because of uncertainty in the denominator. This may be explained by the rather high mobility of this population that, in part, did not necessarily remain in France for a long time. In addition, among the displaced population, it is possible that those who underwent CXR screening may have been at higher risk of being affected with TB, either because they were more often symptomatic or because they more frequently had a history of TB than the general displaced population. Although data are missing in our country to confirm this hypothesis, such a bias may have underestimated the NNS, which was 862 in our survey. This number is, however, lower than the mean NNS of 1,027 reported in a recent literature review when screening adult migrants with TB symptoms in low/moderate TB incidence settings [10]. Even if our screening data overestimate the true TB prevalence in this displaced population, we feel that our programmatic data show that active screening allowed the detection of additional cases that otherwise may have been overlooked.

Our experience also illustrates some difficulties faced for TB control in case of massive population displacement. Firstly, only a small number of the displaced persons were actively screened for TB. Barriers to screening may be linked to different factors such as competing interests, perceived health or scepticism towards the programme purposes [15]. In France, displaced persons were offered accommodation in short-stay community centres (e.g. schools, gymnasiums, hotels) located near entering sites (e.g. borders, train stations, airports) before being offered a more stable accommodation and put in contact with public services and associations. Secondly, families or individuals were encouraged to offer private housing. This strategy of short-stay centres and rapid relocation makes it difficult to offer screening in a timely manner. Thirdly, few persons had TB symptoms or a previous history of TB and therefore most were hesitant to attend a health centre for CXR screening. This is supported by the data from the Strasbourg TB centre, where less than half of the persons referred for screening accepted a CXR. Finally, the priorities of this non-symptomatic population may have been centred towards emergency needs for housing, job or school seeking more than for health issues.

We focused our report on active TB disease, although diagnosis and chemoprophylactic treatment of latent TB infection is another issue in the displaced populations. Active screening for TB of displaced populations has been implemented in other European countries, where the target population was selected through different strategies such as the results of a skin test [16] or the presence of symptoms [17]. The effectiveness of different screening strategies has been evaluated, yielding varying results [18-20]. Our data, albeit from a single country, may somewhat challenge the WHO/ECDC recommendation of not recommending universal screening of refugees arriving in European countries from Ukraine [21].

## Conclusions

Our results underline the interest of a pre-established well-organised network of TB centres such as the CLAT network in case of sudden mass migration from a high TB incidence country. Increasing effectiveness of the existing strategy may require further well-trained manpower and financial support, both likely not being readily available. Although screening strategies could vary in other European countries according to local settings and available resources, it appears of interest evaluating the interventions addressing TB control among displaced populations from Ukraine to adapt international recommendations on observational data.

## References

[1] Council of the European Union. Ukraine: Council unanimously introduces temporary protection for persons fleeing the war. Press release. 4 Mar 2022. Available from: https://www.consilium.europa.eu/en/press/press-releases/2022/03/04/ukraine-council-introduces-temporary-protection-for-persons-fleeing-the-war/

[2] Ministère de l’Intérieur. Instruction relative à la mise en œuvre de la décision du Conseil de l'Union européenne du 5 mars 2022, prise en application de l'article 5 de la directive 2001/55/CE du Conseil du 20 juillet 2001. [Instruction on the implementation of the Decision of the Council of the European Union of 5 March 2022 pursuant to Article 5 of Council Directive 2001/55/EC of 20 July 2001]. Paris: Journal officiel de la République française; 2022. French. Available from: https://www.legifrance.gouv.fr/circulaire/id/45302?page=1&pageSize=10&query=+INTV2208085J&searchField=ALL&searchType=ALL&tab_selection=all&typePagination=DEFAULT

[3] European Centre for Disease Prevention and Control (ECDC). Tuberculosis surveillance and monitoring in Europe 2021 – 2019 data. Stockholm: ECDC; 2021. Available from: https://www.ecdc.europa.eu/en/publications-data/tuberculosis-surveillance-and-monitoring-europe-2021-2019-data

[4] Dahl V Migliori GB Lange C Wejse C . War in Ukraine: an immense threat to the fight against tuberculosis. Eur Respir J. 2022;59(4):2200493. 10.1183/13993003.00493-2022 35450921

[5] Vignier N Halley des Fontaines V Billette de Villemeur A Cazenave-Roblot F Hoen B Chauvin F Public health issues and health rendezvous for migrants from conflict zones in Ukraine: A French practice guideline. Infect Dis Now. 2022;52(4):193-201. 10.1016/j.idnow.2022.04.006 35483634 PMC9040487

[6] Haut conseil de la santé publique (HCSP). Avis relatif aux recommandations concernant la visite médicale des étrangers primo-arrivants en provenance de pays tiers. [Medical examination of newly arrived foreigners]. Paris: HCSP. [Accessed: 14 Feb 2023]. French. Available from: https://www.hcsp.fr/Explore.cgi/avisrapportsdomaine?clefr=672

[7] Haut conseil de la santé publique (HCSP). Avis relatif à la détermination d’un seuil pratique pour définir un pays de haute endémicité tuberculeuse. May 18 2018. [Determination of a threshold of high tuberculosis endemicity]. Paris: HCSP. [Accessed: 14 Feb 2023]. French. Available from: https://www.hcsp.fr/explore.cgi/avisrapportsdomaine?clefr=668

[8] Lailler G Comolet T Riva E Combroure JC . Les activités de maîtrise de la tuberculose menées par les Centres de lutte antituberculeuse. Bilan en 2016 et perspectives. Bull Epidemiol Hebd (Paris). 2018; (6-7):121-4. Available from: http://invs.santepubliquefrance.fr/ beh/2018/6-7/2018_6-7_4.htm

[9] Ministère des solidarités et santé. Instruction DGS/SP2/2020/224 du 8 décembre 2020 relative à la mise en place de la réforme des centres de lutte anti tuberculeuse (CLAT). [Instruction DGS/SP2/2020/224 of 8 December 2020 on the implementation of the reform of tuberculosis control centres (CLAT)]. Paris: Journal officiel de la République française; 2021. French. Available from: https://www.legifrance.gouv.fr/circulaire/id/45119?tab_selection=circ&searchField=ALL&query=*&page=1&init=true&dateSignature

[10] Naufal F Chaisson LH Robsky KO Delgado-Barroso P Alvarez-Manzo HS Miller CR Number needed to screen for TB in clinical, structural or occupational risk groups. Int J Tuberc Lung Dis. 2022;26(6):500-8. 10.5588/ijtld.21.0749 35650693 PMC9202999

[11] Guthmann JP Laporal S Lévy-Bruhl D . La tuberculose maladie en France en 2018. Faible incidence nationale, forte incidence dans certains territoires et groupes de population. Bull Epidemiol Hebd (Paris). 2020; (10-11):196-203. Available from: http://beh.santepubliquefrance.fr/beh/2020/10-11/2020_10-11_1.html

[12] Institut national de la statistique et des études économiques (INSEE). Étrangers par nationalité détaillée. Recensement de la population 2019. [Foreigners and immigrants in 2019. Detailed nationalities and countries of birth - Census of Population]. Paris: INSEE. [Accessed: 20 Mar 2023.] French. Available from: https://www.insee.fr/fr/statistiques/6478065?sommaire=6478362

[13] United Nations Refugee Agency (UNHCR). Operational data portal. Ukraine refugee situation. Geneva: UNHCR. [Accessed: 14 Feb 2023]. Available from: https://data.unhcr.org/en/situations/ukraine#_ga=2.14069111.1340351102.1675866931-785840434.1675866931

[14] Fiebig L Hauer B Andrés M Haas W . Tuberculosis screening in asylum seekers in Germany, 2015: characteristics of cases and yield. Eur Respir J. 2017;50(4):1602550. 10.1183/13993003.02550-2016 29074541

[15] Spruijt I Haile DT Erkens C van den Hof S Goosen S Ten Kate A Strategies to reach and motivate migrant communities at high risk for TB to participate in a latent tuberculosis infection screening program: a community-engaged, mixed methods study among Eritreans. BMC Public Health. 2020;20(1):315. 10.1186/s12889-020-8390-9 32164637 PMC7068882

[16] Solomos Z Botsi C Georgakopoulou T Lytras T Tsiodras S Puchner KP . Active case finding of pulmonary TB in a European refugee camp: lessons learnt from Oinofyta hosting site in Greece. Trop Med Int Health. 2021;26(9):1068-74. 10.1111/tmi.13626 33991376

[17] Pampaloni A Locatelli ME Venanzi Rullo E Alaimo S Cosentino F Marino A "Diagnosis on the Dock" project: A proactive screening program for diagnosing pulmonary tuberculosis in disembarking refugees and new SEI model. Int J Infect Dis. 2021;106:98-104. 10.1016/j.ijid.2021.03.032 33737130

[18] Wahedi K Biddle L Bozorgmehr K . Cost-effectiveness of targeted screening for active pulmonary tuberculosis among asylum-seekers: A modelling study with screening data from a German federal state (2002-2015). PLoS One. 2020;15(11):e0241852. 10.1371/journal.pone.0241852 33151980 PMC7644037

[19] Collin SM Wurie F Muzyamba MC de Vries G Lönnroth K Migliori GB Effectiveness of interventions for reducing TB incidence in countries with low TB incidence: a systematic review of reviews. Eur Respir Rev. 2019;28(152):180107. 10.1183/16000617.0107-2018 31142548 PMC9489042

[20] Heuvelings CC de Vries SG Greve PF Visser BJ Bélard S Janssen S Effectiveness of interventions for diagnosis and treatment of tuberculosis in hard-to-reach populations in countries of low and medium tuberculosis incidence: a systematic review. Lancet Infect Dis. 2017;17(5):e144-58. 10.1016/S1473-3099(16)30532-1 28291722

[21] European Centre for Disease Prevention and Control (ECDC). Testing for tuberculosis infection and screening for tuberculosis disease among refugees arriving in European countries from Ukraine. Stockholm: ECDC; 2022. Available from: https://www.ecdc.europa.eu/en/news-events/testing-tuberculosis-infection-and-screening-tuberculosis-disease-among-incoming

